# Developmental Disabilities and Intracranial Abnormalities in Children with Symptomatic Cytomegalovirus and Cochlear Implants

**DOI:** 10.5402/2012/502746

**Published:** 2012-12-13

**Authors:** Catherine K. Hart, Susan Wiley, Daniel I. Choo, Christine Eby, Laura Tucker, Mark Schapiro, Jareen Meinzen-Derr

**Affiliations:** ^1^Division of Pediatric Otolaryngology Head and Neck Surgery, Cincinnati Children's Hospital Medical Center, Cincinnati, OH 45229, USA; ^2^Division of Developmental and Behavioral Pediatrics, Cincinnati Children's Hospital Medical Center, Cincinnati, OH 45229, USA; ^3^Division of Audiology, Cincinnati Children's Hospital Medical Center, Cincinnati, OH 45229, USA; ^4^Division of Neurology, Cincinnati Children's Hospital Medical Center, Cincinnati, OH 45229, USA; ^5^Division of Biostatistics and Epidemiology, Cincinnati Children's Hospital Medical Center, Cincinnati, OH 45229, USA

## Abstract

*Objective*. To examine the association of intracranial radiographic abnormalities and developmental measures with outcomes in children with congenital symptomatic cytomegalovirus (CMV) and cochlear implants (CI). *Design/Methods*. It was a retrospective review of 15 children implanted from 2004 to 2010. Preimplant nonverbal intelligence quotient/developmental quotient (IQ/DQ) and head circumference (HC) were obtained. Computed tomography and magnetic resonance imaging of the brain and post-CI audiometry and language assessments were reviewed. *Results*. Eleven children (73%) had cognitive delay. Most had >1 developmental disability. Median IQ/DQ was 65 (23–90). All had imaging abnormalities. Most imaging abnormalities were in parietal (60%) and temporal (60%) lobes. Children with HC < 5th percentile had poorer median post-CI PTA (38 dB versus 27 dB, *P* = 0.02). Periventricular calcifications were associated with lower receptive (*r*_*b*_ = −0.75, *P* = 0.03) and expressive (*r*_*b*_ = −0.84, *P* = 0.008) language. Because IQ/DQ was associated with periventricular calcifications (*r*_*b*_ = −0.53, *P* = 0.04) and small HC (*r*_*b*_ = −0.73, *P* = 0.002), their relationships with language appear partially driven by IQ/DQ. *Conclusions*. The location of brain abnormalities appears to correlate with worse outcomes after CI. These findings may allow for more accurate counseling of parents regarding anticipated postimplantation performance.

## 1. Introduction

Cytomegalovirus (CMV) is one of the most common congenital viral infections in many regions. In the United States it occurs in 0.5–1% of live births, or approximately 40,000 infants annually [1]. The manifestations of CMV infection cover a broad spectrum ranging from asymptomatic to severe systemic disease resulting in significant morbidity and mortality. 90% of infants with congenital CMV are asymptomatic at birth. Despite being asymptomatic at birth, up to 7% of these children will develop sensorineural hearing loss (SNHL) that can be unilateral or bilateral, fluctuating or progressive, and range from mild to profound [2, 3]. Approximately 10% of infants with congenital CMV are symptomatic at birth, and 40% of these patients will develop SNHL [1, 2]. Hearing loss is the most common manifestation of congenital CMV infection making CMV a leading cause of nonhereditary congenital hearing loss [4].

Given the relatively large number of children potentially affected by CMV-related hearing loss and the wide range of manifestations of congenital CMV infection, it is difficult to predict how a child with symptomatic CMV will perform with a cochlear implant (CI). Previous studies suggest that children with symptomatic CMV derive auditory benefit from CIs, albeit at a slower rate than other children. The degree to which these children benefit remains somewhat controversial as there is variation in the development of language skills after CI [5–9]. The objective of our study was to examine the relationships between intracranial radiographic abnormalities and preimplantation developmental assessment (including head circumference) with postimplant audiometric and language outcomes in children with symptomatic congenital CMV who have undergone cochlear implantation.

## 2. Materials and Methods

This study was a retrospective review of children with a diagnosis of symptomatic congenital CMV who underwent cochlear implantation between 2004 and 2010 at a tertiary pediatric cochlear implant center. Congenital CMV was diagnosed for most children via urine culture of the virus during the first two weeks of life. Diagnosis was also made based on the identification of hearing loss coupled with brain image findings. Medical charts of these patients were reviewed for pre- and postimplantation evaluations and central nervous system imaging findings. Data regarding preimplantation developmental assessments included the diagnoses of developmental delay and/or other disabilities, nonverbal intelligence quotients or developmental quotients (IQ/DQ), and head circumference (HC) at the preoperative visit. Results from computed tomography (CT) and/or magnetic resonance imaging (MRI) scans of the brain were reviewed, and the findings were classified by type and location of anomaly. Postimplant audiometric behavioral testing, which was performed using validated and developmentally appropriate testing techniques, was collected. This data included frequency-specific thresholds and speech awareness or speech reception thresholds (SAT/SRT). Pure tone averages (PTA) were calculated by an average of 4 frequencies (0.5, 1.0, 2.0, and 4.0 kHz). 

Children's developmental disability diagnoses were classified according to the areas of cognition, motor, vision, and language. Cognitive disability was diagnosed using nonverbal cognitive measures such as the Leiter International Performance Scale [10] and supported by evaluating adaptive functioning with the Vineland Adaptive Behavior Scale [11]. These evaluations were completed by psychologists with knowledge of testing deaf/hard of hearing children. If the nonverbal IQ was not available, a nonverbal developmental quotient was used. This nonverbal or adaptive quotient was obtained from a developmental pediatrician with expertise in hearing loss as a part of the precochlear implant assessment. For this study, a nonverbal IQ/DQ was used. The diagnosis of cerebral palsy (CP) was made based on neurologic examination, patterns of persistent primitive reflexes, abnormalities in tone and reflexes, and presence of abnormalities on MRI of the brain. The diagnosis of pervasive developmental disorder not otherwise specified was made by interdisciplinary evaluations with speech pathology and psychology using standardized measures to evaluate autism spectrum disorders. Standardized language assessment scores, using the Preschool Language Scale (4th edition) [12] were available on eight subjects who returned for speech therapy after implantation. This study was approved by the Institutional Review Board at Cincinnati Children's Hospital Medical Center. 

Data distributions were reported using medians with ranges (for continuous data) and frequencies with percentages (for categorical data). Characteristic differences between children with HC < 5th percentile and >5th percentile were tested using Wilcoxon Rank Sums test for continuous data and chi-square or Fisher's Exact test for categorical data. Biserial correlations were conducted to understand the association between presence and location of brain abnormalities with audiometric and language outcomes. The Wilcoxon Rank Sums test was also used to explore statistical differences regarding factors (e.g., IQ/DQ) that may be associated with presence and location of brain abnormalities. Due to the small sample size, exact *P* values are reported.

## 3. Results

### 3.1. Subject Characteristics

Table 1 lists the fifteen CMV-positive children (9 females and 6 males) who received a CI during the study timeframe. Two additional subjects with CMV were excluded from this analysis: one subject had not returned for any postimplant visits and one child had normal CNS imaging (thought not to be symptomatic CMV). The median age at time of identification of hearing loss was 5 months (range 1–62 months), and the median age at implantation was 28 months (range 12–96 months) (Table 2). The median IQ/DQ for the study population was 65 (range 23–90); 9 (60%) children had an IQ/DQ < 70. Seventy-three percent (*n* = 11) of children had a diagnosis of cognitive impairment and 13 children (87%) had a motor delay or disability; 9 had a diagnosis of CP. Over half (60%, *n* = 9) of these children carried a diagnosis of more than one developmental disability and nearly half (47%, *n* = 7) had both a cognitive disability, and CP. 

### 3.2. Head Circumference < 5th Percentile

Seven children (47%) had a head circumference (HC) less than the fifth percentile (Table 2). Children with a HC < 5th percentile had a significantly lower median IQ/DQ than children with a HC ≥ 5th percentile (42 versus 77, *P* = 0.003). All children with HC < 5th percentile had cognitive disabilities compared to only 50% of children with a HC ≥ 5th percentile. Additionally, the children with a HC < 5th percentile had slightly poorer postimplant PTA (38 dB versus 27 dB, *P* = 0.02) and SAT/SRT (30 dB versus 23 dB, *P* = 0.04) compared to children with larger heads.

### 3.3. Imaging Findings

All children included in the study had central nervous system abnormalities present on imaging studies (Table 3). Twelve children had MRI imaging (all with identified abnormalities) and 13 CT imaging of the brain with identified abnormalities. The majority had more than one abnormality. Eighty percent (*n* = 12) had calcifications, which occurred most frequently (53%) in the periventricular region (Table 4). Additional imaging findings included ventriculomegaly in 53%, migrational abnormalities in 33% (*n* = 5), and encephalomalacia in 27%. Three children had cerebellar abnormalities. Most children had more than one abnormal CNS finding.

All four children with encephalomalacia and all 3 children with cerebellar abnormalities had a diagnosis of CP. In addition to CP, 3 of the 4 children with encephalomalacia and all 3 children with cerebellar abnormalities also had a cognitive disability diagnosis. In fact, the children with cerebellar abnormalities had a lower nonverbal IQ/DQ than children without cerebellar abnormalities (35 (range 23–50) versus 68 (range 27–90), *P* = 0.048). These 3 children had a post-CI PTA ranging from 38 to 57. Location of the calcifications appeared to play a role in audiologic outcomes. Calcifications in the temporal lobes were associated with poorer post-CI PTA (biserial correlation *r*_*b*_ = 0.57, *P* = 0.04). 

### 3.4. Associations with Language

Eight children had postimplant standardized language assessments available. The median (range) receptive language score was 21 (3–27), and expressive language score was 28 (17–77). Two additional children had only prelinguistic language skills postimplant. The IQ/DQ was highly correlated with both receptive (Spearman rho = 0.9, *P* = 0.002) and expressive (Spearman rho = 0.86, *P* = 0.007), as having a head circumference <5th percentile (point biserial correlation *r*_*b*_ = −0.75 and −0.68 resp.). Periventricular calcifications were associated with lower receptive language (*r*_*b*_ = −0.75, *P* = 0.03) and expressive language (*r*_*b*_ = −0.84, *P* = 0.008) among children with post-CI assessments. Because the IQ/DQ was associated with both periventricular calcifications (*r*_*b*_ = −0.53, *P* = 0.04) and HC < 5th percentile (*r*_*b*_ = −0.73, *P* = 0.002), the relationship between these factors and language appears to be, at least in part, driven by IQ/DQ scores.

## 4. Discussion

One of the challenges in children with congenital CMV is predicting the sequelae they will develop and the extent of neurological and developmental deficits they will incur. Previous studies have shown that brain imaging may be a good predictor of adverse neurodevelopmental outcomes. In one study of children with symptomatic congenital CMV, 90% of the children with an abnormal CT scan developed at least one sequela compared to 29% of those with a normal scan [13]. Greater than two-thirds of all children with congenital CMV had MRI abnormalities and, of these, cortical malformations, ventriculomegaly and hippocampal dysplasia correlated highly with poor neurologic outcome [14]. The presence of microcephaly and calcifications are also indicative of poor neurologic outcome [15]. Noyola et al. found that microcephaly was the most specific predictor, and abnormality on head CT was the most sensitive predictor of poor cognitive outcomes in this patient population [16]. In a study of children with a diagnosis of SNHL, 80% of the CMV positive children had abnormal brain MRI scans compared with only 33% of CMV negative children [17]. Our study demonstrated that certain imaging findings may correlate with worse outcomes after CI. Children with calcifications had a slightly poorer median post-CI PTA compared to children without calcifications. Interestingly, the location of the abnormalities also seemed to correlate with worse language outcomes. The majority of the abnormalities were found in the parietal lobe and temporal lobe. The parietal lobe processes sensory information and houses our language abilities, and the temporal lobe regulates emotion, hearing, language, and learning, which could explain why language outcomes are poorer in children with abnormalities in these regions. Children with cerebellar anomalies also had lower receptive and expressive language levels than the other children. Although the cerebellum has been largely thought to control balance, there may be other aspects of learning and development which depend on this entry point of sensory information to the higher cortical areas. The authors have implicated the cerebellum to be involved in temporal processing, language production and comprehension, spatial reasoning, and visual attention [18–21]. In our 3 patients with cerebellar lesions, it is not clear if it is the location of the lesions in the cerebellum or the severe cognitive delay that resulted in worse outcomes. With only 3 patients, it is not possible to draw additional conclusions regarding cause and effect in this group. 

In addition to looking at the associations between CNS imaging findings and post-CI outcomes, our study also looked for correlations between preimplantation developmental assessment and cochlear implant performance in these patients. Although there is a relative paucity of relevant studies in the literature, previous studies have demonstrated that children with cognitive delay seem to derive benefit from CI, albeit at a slower rate than nondelayed children [22, 23]. Although children with cochlear implants who have more significant developmental delay have poorer outcomes than children with only mild delay [24], the biggest predictor of language development among children with implants and disabilities is a measure of nonverbal cognitive ability [25]. Unfortunately, when compared to appropriately matched developmentally delayed children without hearing impairment, children with CIs had lower receptive and expressive language skills, which were not commensurate with their cognitive potential [26]. Although children with multiple handicaps may progress slower and to a lesser extent than typically developing children, these children still derive benefit from the auditory stimulation provided by a CI but may require additional post-CI intervention to optimize benefit [27–29]. 

As we found in our study, the majority of children with symptomatic congenital CMV have associated developmental disabilities. The variability of neurocognitive deficits in these children warrants the examination of this group separately from other children with developmental disabilities. As with children with developmental disabilities, children with symptomatic congenital CMV appear to derive benefit from CI albeit at a slower rate. Ramirez Inscoe and Nikolopoulos demonstrated mixed results for speech perception and intelligibility with 50% of children with congenital CMV performing more poorly than controls, 31% performing similarly, and 19% performing better than controls [5]. These children did, however, derive auditory benefit from CI. In their study of 13 children with symptomatic congenital CMV, 73% of implanted children achieved closed-set word recognition, and 63% achieved open-set word recognition [9]. Children with congenital CMV have also demonstrated both improved pure-tone hearing thresholds and improved language perception and production [6] as well as useful speech comprehension albeit lower than matched controls [7]. Another study of 11 children with congenital CMV found that 9 of the 11 carried a diagnosis of psycho-neurological disorders (including attention deficit hyperactivity disorder, mental retardation, autism, and pervasive developmental disorder). Although hearing thresholds were similar among children in this group, post-CI performance varied widely depending upon the concomitant diagnosis [8]. Our findings were consistent with these previous studies in that our patients did demonstrate overall improvement in post-CI thresholds. 

Our results indicate that head size was significantly related to IQ/DQ and that children with microcephaly (HC < 5th percentile) were more likely to have cognitive disabilities than those without microcephaly. The microcephalic children also had significantly poorer post-CI PTA and SAT/SRT as well as worse receptive language. These findings indicate that microcephalic children do not achieve the same degree of auditory benefit as the children with normal head size. The mean age at last audiometric followup did vary between the microcephalic and nonmicrocephalic groups (46 versus 59 months). This difference would not be expected to affect the results of audiometric testing as all testing was performed using developmentally appropriate behavioral methods that should account for the difference in age. Given that the majority of the children (73%) had developmental delay and nearly half were considered multiply handicapped, it is notable that head circumference was a better predictor of post-CI outcome than specific developmental delay diagnosis. This is not entirely surprising as head circumference relates to brain growth, which can impact developmental outcomes [30]. 

## 5. Conclusions

Although our study has limitations including its retrospective nature and small sample size, it provides data that may further efforts to identify factors which may help predict which children with congenital symptomatic CMV will benefit from CI. The presence of cerebellar anomalies or greater than one CNS abnormality on imaging correlates with poorer outcomes after CI. The location of CNS abnormalities, including calcifications, may play a role in audiometric and language outcomes after CI. Early measurements such as brain imaging findings, head circumference, and IQ/DQ may allow for more accurate counseling of families regarding anticipated postimplantation performance in children with symptomatic congenital CMV. 

## Figures and Tables

**Table 1:** Fifteen children with symptomatic congenital cytomegalovirus who received cochlear implants.

Subject	Age at CI	Age at ID	Cognitive impairment	Motor impairment	Visual impairment	CI PTA	HC
1	12 m	1 m	Yes	Yes, CP	No	Cnt	5th–50th
2	13 m	2 m	No*	Yes, CP	No	32	>50th
3	16 m	2 m	Yes	Yes	No	25	5th–50th
4	17 m	2 m	Yes	Yes, CP	Yes	33	<5th
5	21 m	15 m	Yes	Yes	No	32	<5th
6	23 m	1 m	Yes	Yes, CP	Yes	57	<5th
7	26 m	11 m	Yes	Yes, CP	No	28	>50th
8	28 m	23 m	Yes	Yes, CP	Yes	38	<5th
9	29 m	5 m	Yes	No	No	27	<5th
10	30 m	5 m	Yes	Yes, CP	Yes	38	<5th
11	38 m	3 m	Yes	Yes	No	80	<5th
12	40 m	13 m	No	Yes	No	33	5th–50th
13	60 m	54 m	No*	No	No	Cnt	>50th
14	86 m	62 m	Yes	Yes, CP	No	23	5th–50th
15	96 m	60 m	No	Yes, CP	No	20	5th–50th

*Had a diagnosis of an autism spectrum disorder.

CI: cochlear implant; ID: identification (of hearing loss); PTA: pure tone average, HC: head circumference; m: months; cnt: could not test; CP: cerebral palsy.

**Table 2:** Summary of subject characteristics.

Characteristics	*N* (%) or median (range) [IQR]	HC < 5th percentile *n* = 7 (47%)	HC > 5th percentile *n* = 8
Age at identification (months)	5 (1–62) [2–23]	5 (1–23)	12 (1–62)
Median IQ/DQ	65 (23-90) [42–77]	42 (23–70)^†^	77 (50–90)
Age at CI surgery (months)	28 (12–96) [17–40]	28 (17–38)	33 (12–96)
Bilateral cochlear implants	3 (20%)	0	3
Full insertion	13 (87%)	6	7
Number of activated electrodes*	21 (10–22) [16–22]	18 (10–22)	22 (10–22)
Age at last audio follow-up	55 (32–169) [40–84]	46 (32–110)	59 (34–169)
Post-CI pure tone average**	32 (20–80) [27–38]	38 (27–80)^†^	27 (20–33)
Post-CI SAT or SRT**	25 (15–80) [23–30]	30 (25–80)^†^	23 (15–25)

*9 children had ≥20 activated electrodes.

**Two children could not complete post-CI audiologic testing at time of study.

^†^ *P* ≤ 0.05 compared with HC ≥ 5th percentile.

CI: cochlear implant; HC: head circumference; IQR: interquartile range; SAT: speech awareness threshold; SRT: speech recognition threshold.

**Table 3:** Central nervous system (CNS) imaging findings.

Types of CNS abnormalities	*N*	%*
Calcifications	12	80%
Ventriculomegaly	8	53%
Migrational abnormalities	5	33%
Encephalomalacia	4	27%
Cerebellar abnormalities	3	20%
White matter loss	1	7%
White matter changes	1	7%
Total number of abnormal findings		
1	3	20%
2	7	47%
3	3	20%
4	2	13%

*Adds up to >100% as children had more than one type of CNS imaging abnormality.

**Table 4:** Location of CNS abnormalities.

Location	*N*	%
Calcifications		
Periventricular	8	53%
Temporal lobe	6	40%
Frontal lobe	6	40%
Frontal horn	2	13%
Parietal lobe	2	13%
Occipital horn	1	7%
Cerebellum	1	7%

Abnormalities		
Parietal lobe	9	60%
Temporal lobe	9	60%
Frontal lobe	7	47%
Cerebellum	2	13%
Cerebral hemisphere	2	13%
Occipital lobe	1	7%
